# Diffractaic acid exerts anti-cancer effects on hepatocellular carcinoma HepG2 cells by inducing apoptosis and suppressing migration through targeting thioredoxin reductase 1

**DOI:** 10.1007/s00210-024-02980-5

**Published:** 2024-02-03

**Authors:** Emine Karaca Sulukoğlu, Şükran Günaydın, Şeyda Nur Kalın, Ahmet Altay, Harun Budak

**Affiliations:** 1 Science Faculty, Department of Molecular Biology and Genetics, Atatürk University, 25240 Erzurum, Turkey; 2 Faculty of Science, Department of Molecular Biology and Genetics, Erzurum Technical University, 25100 Erzurum, Turkey; 3 East Anatolia High Technology Application and Research Center, Atatürk University, 25240 Erzurum, Turkey; 4 Faculty of Engineering and Natural Sciences, Department of Molecular Biology and Genetics, Kütahya Health Sciences University, 43100 Kütahya, Turkey; 5 Faculty of Science and Arts, Department of Chemistry, Erzincan Binali Yıldırım University, 24100 Erzincan, Turkey

**Keywords:** Diffractaic acid, Hepatocellular carcinoma, Cytotoxicity, Thioredoxin reductase 1, Inhibition, Expression

## Abstract

Hepatocellular carcinoma (HCC) represents one of the most common malignant tumors worldwide. Due to the limited number of available drugs and their side effects, the development of new chemotherapeutic strategies for HCC treatment has become increasingly important. This study is aimed at investigating whether diffractaic acid (DA), one of the secondary metabolites of lichen, exhibits a potential anticancer effect on HepG2 cells and whether its anticancer effect is mediated by inhibition of thioredoxin reductase 1 (TRXR1), which is a target of chemotherapeutic strategies due to overexpression in tumor cells including HCC. XTT assay results showed that DA exhibited strong cytotoxicity on HepG2 cells with an IC_50_ value of 78.07 µg/mL at 48 h. Flow cytometric analysis results revealed that DA displayed late apoptotic and necrotic effects on HepG2 cells. Consistent with these findings, real-time PCR results showed that DA did not alter the BAX/BCL2 ratio in HepG2 cells but upregulated the P53 gene. Moreover, the wound healing assay results revealed a strong anti-migratory effect of DA in HepG2 cells. Real-time PCR and Western blot analyses demonstrated that DA increased TRXR1 gene and protein expression levels, whereas enzyme activity studies disclosed that DA inhibited TRXR1. These findings suggest that DA has an anticancer effect on HepG2 cells by targeting the enzymatic inhibition of TRXR1. In conclusion, DA as a TRXR1 inhibitor can be considered an effective chemotherapeutic agent which may be a useful lead compound for the treatment of HCC.

## Introduction

Hepatocellular carcinoma (HCC) is one of the most common malignancies worldwide and is an important subtype of primary liver cancer with an incidence rate of 90% (Cheng et al. 2016; Sung et al. 2021). Survival rates in HCC patients are quite low and generally have 5-year survival rates (Luo et al. 2017). The treatment methods developed for HCC are used to prolong survival and improve quality of life (Chabner et al. 2007; Gao et al. 2021). Among these therapies, systemic chemotherapy continues to play a leading role in the treatment of HCC despite its high side effects. Currently, commercial chemotherapeutic agents such as sorafenib and lenvatinib are the mainstay of HCC treatment, but some natural products are under clinical evaluation. Moreover, these natural products such as icaritin, ginsenoside Rg3, and irinotecan have been recognized as potent chemotherapeutic agents for HCC due to their effects on oxidative stress, angiogenesis, and metastasis (Man et al. 2021). However, due to the limited number of these natural compounds, new chemotherapeutic agents need to be discovered.

Lichens, one of the natural sources of compounds, produce many primary and secondary metabolites. Secondary lichen metabolites have a large spectrum of biological functions such as antiproliferative, antimicrobial, antiviral, antimycotic, antiparasitic, anesthetic, and anti-inflammatory effects (Cetin Cakmak and Gülçin 2019; Cimmino et al. 2019; Kalın et al. 2022b). Diffractaic acid (DA), one of the lichen secondary metabolites, shows antioxidant, immunostimulant, analgesic, and antipyretic effects (White et al. 2014). Its anticancer activity is also demonstrated against cancer cells, including human lung, breast, and epithelial cancers (Truong et al. 2014; Kalın et al. 2022a; Günaydın et al. 2023). However, it still remains unclear whether DA has anti-cancer activity in human liver cancer.

Inflammatory mechanisms and oxidative stress underlie the pathophysiology of HCC (D’souza et al. 2020). Oxidative stress is a condition that occurs when ROS levels in the cell are not kept in balance by antioxidant defense systems (Sosa et al. 2013). As a result, excessive levels of ROS in the cell lead to redox imbalance and contribute to the pathogenesis of various diseases, including cancer, neurodegenerative, and cardiovascular diseases (Kim et al. 2015; Kirtonia et al. 2020). In cancer cells, in addition to ROS, antioxidant defense systems such as the thioredoxin (Trx) system increase their activity to cope with it. Moreover, in cancer cells, the Trx system contributes to tumor development and progression through many different physiological processes including cell proliferation, apoptosis, and metastasis (Jia et al. 2019).

The Trx system plays a versatile role in cells and consists of thioredoxin reductase (TRXR), thioredoxin (TXN), and nicotinamide adenine dinucleotide phosphate (NADPH) (Mahmood et al. 2013; Sönmez Aydın et al. 2021). TRXR has three isoforms in the cell: cytosolic (TRXR1), mitochondrial (TRXR2), and testis-specific (TGR) (Arnér 2017). In most cancers, TRXR1 is expressed at extremely high levels (Yoo et al. 2006; Jia et al. 2019; Kalın et al. 2023). Therefore, a growing body of literature suggests that TRXR1 may be a diagnostic marker for many cancers, including HCC (Branco et al. 2014; Wu et al. 2021). On the other hand, it has also been shown that TRXR1 may be an important target for chemotherapeutic agents in cancer studies (Ozgencli et al. 2018a, b). The aim of this study was to investigate the in vitro anticancer effects of DA on hepatocellular carcinoma (HepG2) cells and to determine whether these potential effects of DA are mediated by targeting TRXR1.

## Material and methods

### Cell culture

The hepatocellular carcinoma (HepG2) cancer cell line was purchased from ATCC (American Type Culture Collection, LGC Promochem, UK). The cells were grown in DMEM (Dulbecco’s Modified Eagle Medium, Sigma-Aldrich), which was supplemented with 10% (v/v) heat-inactivated fetal bovine serum (FBS) (HyClone, Logan, UT, USA), 1% penicillin/streptomycin (Thermo Fisher Scientific), and 1% L-glutamine (Thermo Fisher Scientific) and maintained in a carbon dioxide incubator (5%) at 37 °C.

### Preparation of diffractaic acid (DA)

DA (C_20_H_22_O_7_) was purchased from TargetMol Chemicals Inc. (Boston, MA, USA). A stock solution was prepared by dissolving the DA in dimethyl sulfoxide (DMSO) and stored at − 20 °C. DA was diluted with freshly prepared complete DMEM medium, and serial dilutions were performed to obtain concentrations of 10–250 μg/mL.

### Cytotoxicity assay

The cytotoxic effect of DA on HepG2 cells was assessed by XTT (sodium 3′-[1- (phenylamino carbonyl)- 3,4- tetrazolium]-bis (4-methoxy6-nitro) benzene sulfonic acid hydrate) assay (Cell Proliferation Kit, Roche). Accordingly, the cells were seeded in 96-well plates at a density of 1 × 10^4^ cells/well in 200 μL culture medium per well and incubated overnight in a CO_2_ incubator. Then, the cells were treated with varying concentrations of DA (10–250 μg/mL) in a time-dependent (24 and 48 h) manner. After the incubation periods, the old medium was removed, 100 μL fresh medium was added, and 50 μL of XTT solution was placed in each well. After the incubation for 7 h in a CO_2_ incubator, the absorbance of the wells was measured colorimetrically at 470 nm using an Epoch microplate reader (BioTek, USA). The results were expressed with IC_50_ values (the concentration reducing the cell viability by 50%) and shown with the standard deviation (± SD) of three independent experiments. The following studies were continued with the lowest IC_50_ value from the XTT experiments, i.e., the most cytotoxic.

### Flow cytometry analysis

To determine the apoptotic effect of DA in HepG2 cells, the Annexin V-FITC/PI double staining assay was carried out using manufacturer’s procedure (BioLegend, San Diego, CA) (Altay et al. 2022). First, the cells were seeded in a 6-well plate at 1.5 × 10^5^ cells/well and incubated in a CO_2_ incubator overnight at 37 °C. Then, the cells were treated with the IC_50_ value (78.07 μg/mL) of DA obtained from 48 h. Following the incubation for 48 h, the cells were collected, washed twice with cold Dulbecco’s phosphate buffered saline (DPBS, Sigma-Aldrich), and suspended in 100 μL of Annexin V binding buffer containing 5 μL Annexin V-FITC and 10 μL propidium iodide. After 15 min incubation at room temperature in the dark, 400 μL Annexin V binding buffer was added to the test tubes. A certain amount of the cell suspension was transferred into 96-well plates. Analyses were performed using a Beckman Coulter Cyto-FLEX flow cytometer (Beckman Coulter, Brea, CA, USA). Untreated cells served as a negative control group. The results represented three independent experiments.

### Wound healing assay

The anti-migratory effect of DA on HepG2 cells was investigated by wound healing assay. For this, the cells were seeded at 5 × 10^5^ cells/well in a 6-well plate and incubated in a CO_2_ incubator. After reaching 90% confluence of the cells, a scratch was made in the center of each well using a sterile pipette tip. After washing the plate with DPBS, complete DMEM medium was added in the absence or presence (78.07 μg/mL) of DA, and the culture was allowed to grow at 37 °C. The changes in wound area on the cells were monitored under an inverted microscope at different time intervals (0, 12, 24, 48, and 72 h) (Bobadilla et al. 2019).

### Quantitative real-time PCR

To determine the quantitative change in expression of the apoptosis pathway genes BAX, BCL2, and P53, and the thioredoxin system gene TRXR1 in HepG2 cells, the cells were treated with the IC_50_ value (78.07 μg/mL) obtained from DA for 48 h, and then RNA isolation (RNA Isolation PureLink™RNA Mini Kit, Invitrogen, Carlsbad, CA, USA), cDNA synthesis (High-Capacity cDNA Reverse Transcription Kit, Applied Biosystems), and real-time PCR analyses (Rotor-Gene Q, Qiagen) were performed, respectively. Quantitative gene expression analysis of the target genes was performed using SYBR Green Master Mix (Bio-Rad, Hercules, CA, USA), and the results were normalized to the housekeeping gene β-actin. Gene expression analyses were carried out using the 2^−ΔΔCT^ method (Livak and Schmittgen 2001).

### Western blotting analysis

The effect of DA on the quantitative protein expression of TRXR1 in HepG2 cells was performed by Western blot analysis. First, HepG2 cells grown in a cell culture petri dish were treated with the IC_50_ value (78.07 μg/mL) of DA and incubated for 48 h. Then, total protein was isolated from the cells according to the protocol of Günaydın et al. (2023), and total protein concentration was determined by Bradford protein assay. Second, 20 μg of protein was separated on SDS polyacrylamide gel (14%). Finally, for Western blot analysis, proteins in the gel were transblotted onto PVDF (polyvinylidene fluoride) membranes and blocked for 1 h. The membrane was then immunoblotted with specific primary antibodies (anti-TRXR1, Santa Cruz Biotechnology, sc-28321; anti-β-Actin, Santa Cruz Biotechnology, sc-47778) at a 1:1000 dilution overnight at 4 °C. After incubation, blots were visualized using a chemiluminescence detection technique (ECL Clarity/ECL Clarity Max Substrate, Bio-Rad) with horseradish coupled secondary antibodies (Santa Cruz Biotechnology, 1:10,000). Band intensity was analyzed using ImageJ2x software (Altun and Budak 2021).

### Analysis of TRXR enzyme activity

DTNB (5,5′-dithiobis-2-nitrobenzoic acid) method was used to determine the effect of DA on TRXR1 enzyme activity in HepG2 cells according to the protocol of Günaydın et al. (2023). First, HepG2 cells grown in a cell culture petri dish were treated with the IC_50_ value (78.07 μg/mL) of DA and incubated for 48 h. Then, total protein was isolated from the cells. TRXR1 enzyme activity was determined by measuring TNB (5-thio-2-nitrobenzoic acid) formed as a result of the reduction reaction of DTNB with NADPH per minute. Changes in TRXR1 enzyme activity were monitored spectrophotometrically at a wavelength of 412 nm every 3 min (Kalın et al. 2022b).

### Statistical analysis

Each set of experiments was performed at least three times, and the results were statistically evaluated using GraphPad Prism Software for Windows version 7.0 (GraphPad Software, LaJolla CA, USA). Two-way analysis of variance (ANOVA) was used for XTT assay and flow cytometry analysis. Unpaired *t*-test was used for quantitative real-time PCR, wound healing assay, western blotting, and TRXR enzyme activity analysis. In Western blotting analysis, band intensity was determined using ImageJ2x software. *p* values less than 0.05 were considered statistically significant, and the symbol (*) indicates statistically significant changes.

## Results

### Diffractaic acid (DA) shows antiproliferative activity on HepG2 cells

**Fig. 1:**
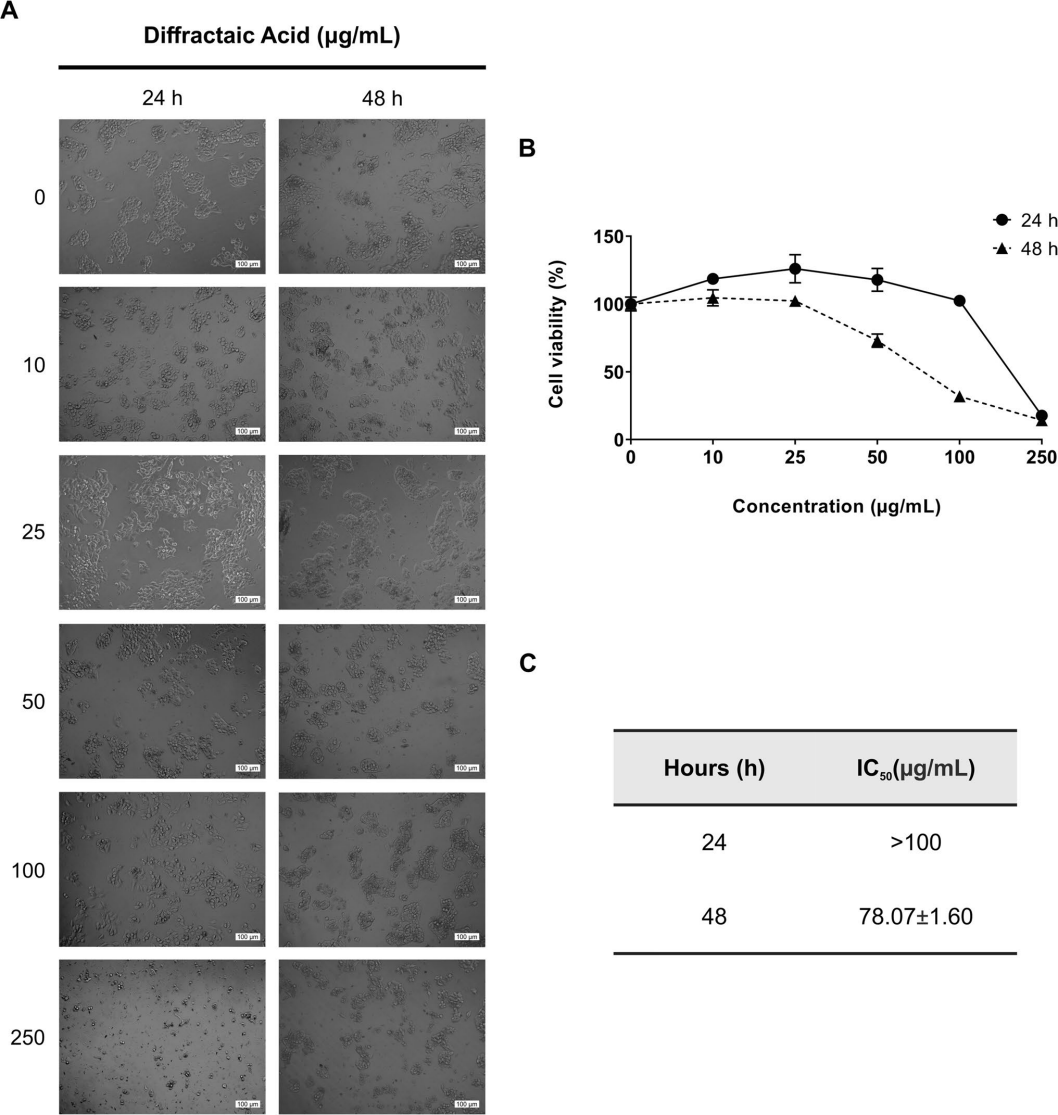
Antiproliferative effect of DA against HepG2 cells. **A** Microscopic images of DA-treated HepG2 cells in a dose (10–250 µg/mL) and time (24 and 48 h) dependent manner.** B** Viability of HepG2 cells assessed by XTT assay after treatment with DA in a dose and time-dependent manner. **C** The IC_50_ values of DA on HepG2 cells. Data are representative of three independent experiments and are presented as means ± SDs. *p < 0.05 (significant), **p < 0.01 (very significant), and ****p < 0.0001 (extremely significant). Scale bar 100 µm

To determine the possible anticancer effect of DA on HepG2 cells, cytotoxicity tests, which are considered first-stage experiments, were performed. Our results showed that treatment of HepG2 cells with different concentrations of DA (10–250 µg/mL) significantly reduced the proliferation compared to the untreated cells for both 24 and 48 h (Fig. 1A, B). The IC_50_ values for DA for both incubation times were calculated as > 100 µg/mL and 78.07 ± 1.60 µg/mL (*p* < 0.0001) at 24 and 48 h, respectively (Fig. 1C). These results reveal that the most effective cytotoxic activity for DA was obtained after 48 h of application.

### Diffractaic acid (DA) induces apoptosis in HepG2 cells

**Fig. 2:**
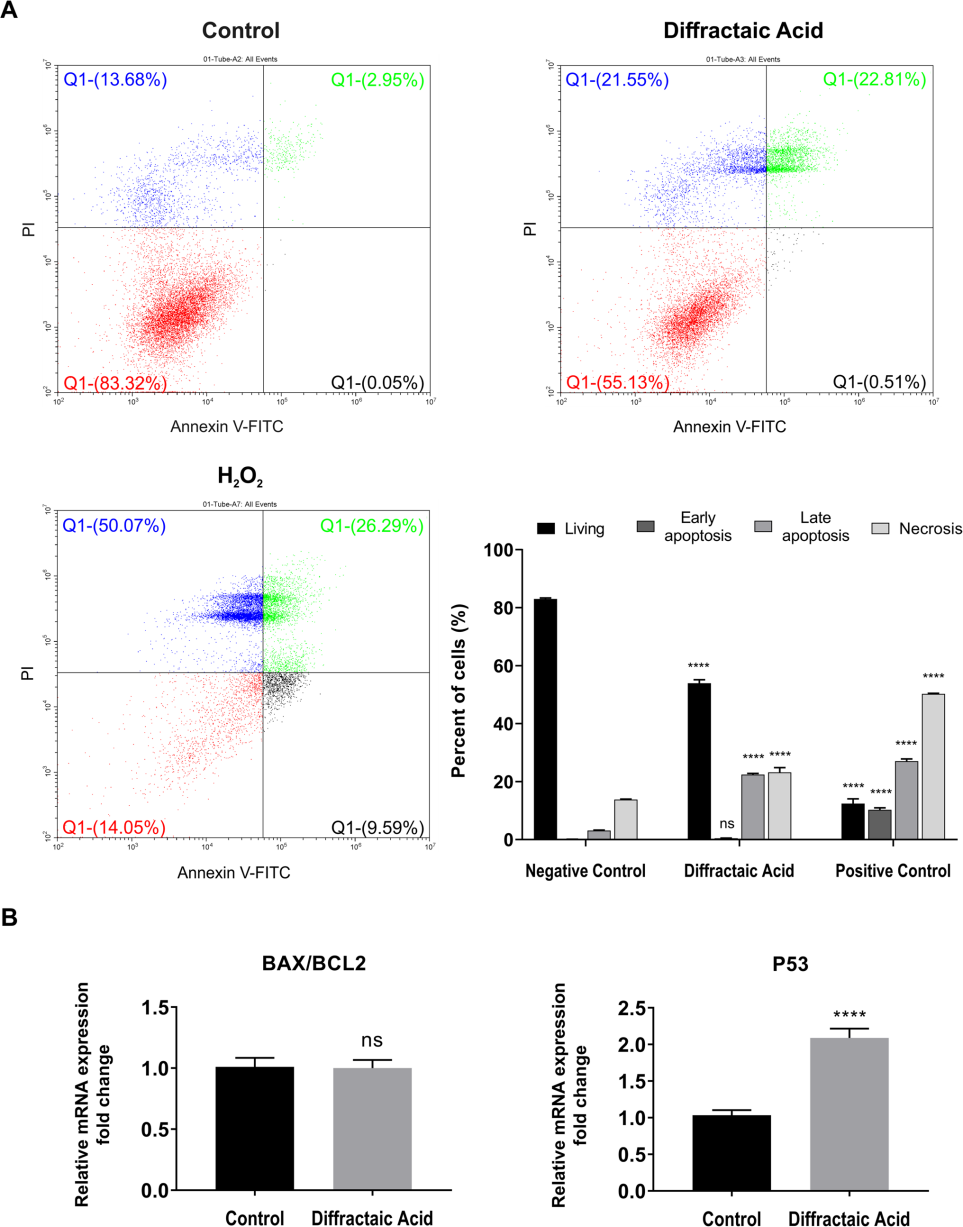
Induction of apoptosis in HepG2 cells by DA. **A** Flow cytometry results of hydrogen peroxide as a positive control (750 µM) and DA-treated HepG2 cells after 48 h incubation time. The living, early apoptotic, late apoptotic, and necrotic cells were represented by the lower left quadrant (Annexin V-FITC-/PI-), lower right quadrant (Annexin V-FITC + /PI-), upper right quadrant (Annexin V-FITC + /PI +), and upper left (Annexin V-FITC-/PI +) quadrant, respectively. **B** Representative bar graphs of BAX/BCL2 ratio and the relative expression of P53 gene in HepG2 cells after treatment with DA for 48 h. Data are representative of three independent experiments and are presented as means ± SEMs. ns *p* > 0.05 (not significant, ns) and ****p < 0.0001 (extremely significant)

To investigate whether the cell viability-reducing effect of DA in HepG2 cells was due to apoptosis, the percentage of apoptotic cells was measured by staining with Annexin V-FITC/PI following the treatment of the HepG2 cells with the IC_50_ value of DA for 48 h. The results showed that there was a significant increase in the population of cells prone to late apoptosis (from approximately 3 to 22%) (*p* < 0.0001) and a 7% increase in the population of cells undergoing necrosis (p < 0.0001), but no significant change in early apoptosis (*p* > 0.05) in HepG2 cells treated with DA compared to the control cells (Fig. 2A).

To confirm the flow cytometry analysis results, the changes in the expression of BAX, BCL2, and P53, which are considered marker genes associated with the apoptotic pathway, were performed with real-time PCR. As shown in Fig. 2B, DA did not cause a significant change in the BAX/BCL2 ratio (*p* > 0.05) but significantly increased the expression of the P53 gene (*p* < 0.0001).

### Diffractaic acid (DA) prevents the migration of HepG2 cells

**Fig. 3:**
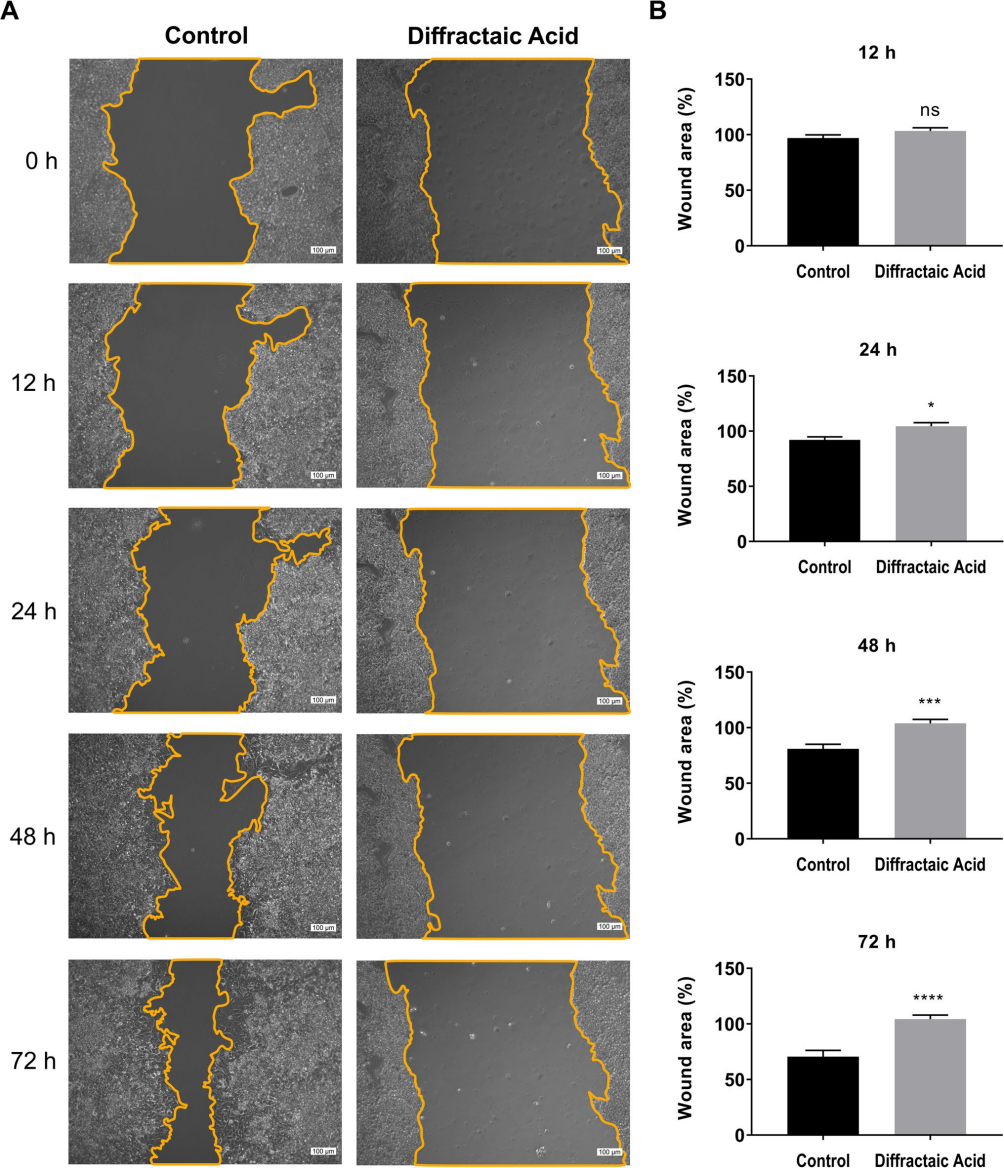
Suppression of migration of HepG2 cells by DA. **A** Representative images and quantitative analysis of wound healing assay in HepG2 cells after treatment with DA. **B** The area of the wound was measured in each well using ImageJ software analysis. Data are representative of the mean of six measurements of each wound area in three independent experiments (*n* = 18) and are presented as mean ± SEMs. *p < 0.05 (significant), ***p < 0.0005 (extremely significant), and ****p < 0.0001 (extremely significant). Scale bar 100 µm

To determine the possible antimigratory effect of DA in HepG2 cells, wound healing assay was performed. For this, the HepG2 cells were treated with the IC_50_ value of DA, and a wound area was created with a pipette tip. Afterward, the closure of the wounded area was monitored at 12, 24, 48, and 72 h, respectively. As seen in Fig. 3A, wound site closure was much faster in control cells compared to the cells treated with DA. Also, the statistical results in Fig. 3B showed that DA inhibited the migration of the cells by 12.30% (*p* < 0.05), 22.95% (*p* < 0.0005), and 33.75% (*p* < 0.0001) at 24, 48, and 72 h, respectively, compared to the control group. These findings clearly indicated that DA has an antimigratory ability against HepG2 cells, in vitro.

### Diffractaic acid (DA) affects thioredoxin reductase 1 at transcriptional, translational, and post-translational levels

**Fig. 4:**
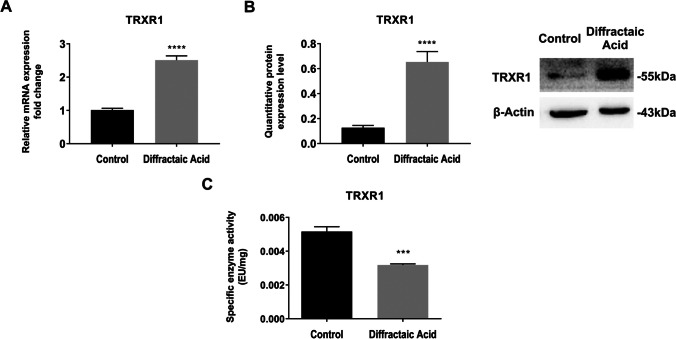
The effect of DA on TRXR1 at gene and protein expressions, and enzymatic activity level in HepG2 cells.** A, B** Changes in quantitative mRNA and protein expression levels of TRXR1 by DA in HepG2 cells. **C** Changes in the enzymatic activity level of TRXR1 by DA in HepG2 cells. Data are representative of three independent experiments and presented as means ± SEMs. ***p < 0.0005 (extremely significant) and ****p < 0.0001 (extremely significant)

The effects of DA on TRXR1, one of the thioredoxin system members, at gene, protein, and enzyme activity levels in HepG2 cells were investigated by real-time PCR, Western blot, and DTNB methods, respectively. The data obtained from Fig. 4A–C showed that DA induced the expression of TRXR1 at gene and protein levels (*p* < 0.0001) while suppressing the enzymatic activity level of TRXR1 (*p* < 0.0005).

## Discussion

Chemotherapy is one of the most preferred cancer treatment methods due to the fact that it can reach all cancerous cells during the treatment process. However, it has some limitations as it can show its effect on healthy cells as well as cancerous cells (Pearce et al. 2017). Therefore, it is important to identify novel natural chemotherapeutic agents with few side effects and cell-specific (Mondal et al. 2012; Gao et al. 2023). Lichen secondary metabolites among these natural products have various biological properties, including anticancer activity (Shrestha et al. 2015). In our previous studies, we showed the potential anticancer activity of DA, a lichen secondary metabolite, in human breast (MCF-7 and MDA-MB-453) and lung (A549) cancer cell lines (Kalın et al. 2022a; Günaydın et al. 2023). However, it has not been reported whether it has anticancer activity against hepatocellular carcinoma (HCC), which is one of the most common malignant tumors in the world and the leading cause of cancer-related deaths.

In this context, we first examined the dose- and time-dependent antiproliferative effect of DA on the hepatocellular carcinoma cancer cell line (HepG2) in vitro. The XTT results showed that DA had a remarkable cytotoxic effect against HepG2 cells with the most effective IC_50_ value of 78.07 µg/mL at 48 h. Several studies have been published on the cytotoxic effects of DA against different cancer cell lines. Brandão et al. (2013) reported that DA showed a cytotoxic effect against UACC-62 and B16-F10 melanoma cells with LC_50_ values of 176.8 μg/mL and 198.2 μg/mL, respectively. In another study, Truong et al. (2014) reported that DA had a potent cytotoxic effect against MCF-7 (human breast cancer), HeLa (human epithelial carcinoma), and NCI-H460 (human lung cancer) cell lines at 89.7 μg/mL, 90.4 μg/mL, and 89.5 μg/mL, respectively. Also, our previous studies revealed that DA exhibited the IC_50_ values of 51.32 μg/mL, 87.03 μg/mL, 46.37 μg/mL, and 22.52 μg/mL on the human breast (MCF-7 and MDA-MB-453), lung (A549), and cervical (HeLa) cancer cell lines, respectively (Kalın et al. 2022a; Günaydın et al. 2023; Budak et al. 2023). Besides, the findings in the literature indicated that DA is cytotoxic against lung and gastric cancers at concentrations up to 100 µg/mL, but does not show any toxicity against HUVEC (healthy human umbilical cord vascular endothelial cells) at concentrations up to 200 µg/mL for 48 h (Kızıl and Ağar 2017). Therefore, it was concluded that the IC_50_ concentration of DA determined against HepG2 cells in this study could not cause a cytotoxic effect on normal cells. These data showed that the cytotoxic effect of DA on different cell lines reported in the literature was also demonstrated against HepG2 cells in this study for the first time. This cytotoxic effect also seems to vary significantly according to the cell type and time of application.

Upon the literature is examined, whether the cytotoxic effect of chemotherapeutic drugs on cancer cells leads the cells to apoptosis is a widely examined situation. Apoptosis, known as programmed cell death, is a physiological process required to eliminate damaged cells. It is regulated by members of the BCL2 family including BAX (pro-apoptotic) and BCL2 (anti-apoptotic), and an increased level of the BAX/BCL2 ratio in the cells induces apoptosis (Tait and Green 2010). In addition, apoptosis can be promoted by tumor suppressors such as P53 in response to ROS-induced DNA damage (Ott et al. 2007). Necrosis, on the other side, is an uncontrolled and accidental cell death that occurs due to factors such as extreme physicochemical stress, overproduction of ROS, and depletion of ATP (Golstein and Kroemer 2007; Krysko et al. 2008). As a common feature in all cancer types, cancer cells develop resistance to apoptosis and continue their survival and proliferation. Therefore, promoting apoptosis has become an important target in cancer therapy (Pfeffer and Singh 2018). The cytotoxic effect of many lichen acids against different cancer types has been determined to be related to apoptosis (Kalın et al. 2022a; Günaydın et al. 2023). When we examined the cause of the cytotoxic effect of DA with flow cytometric analysis in detail, late apoptosis and necrosis were observed. Besides, gene expression analyses supported the flow cytometry results, such that no change in the BAX/BCL2 ratio was observed, suggesting that DA drives cells to late rather than early apoptosis. Moreover, we found that DA caused an increase in the expression levels of the P53 gene in HepG2 cells. This finding has been greatly supported by other studies in this field linking increased P53 levels with late apoptosis and necrosis (Peled et al. 1996; Vaseva et al. 2012).

The tendency to migrate is a natural process for normal cells to grow and maintain proper tissue function but it also leads to invasion and metastasis in cancer cells (Friedl and Wolf 2003). One of the leading causes of cancer-related deaths is metastatic character, and metastasis is observed in almost all cancer types. HCC is one of the primary cancer types with a high recurrence rate and the ability to metastasis to various tissues, especially the lung, lymph nodes, bone, and brain (Chabner et al. 2007). Although several research have been carried out on the anti-invasion and anti-metastatic activity of many lichen secondary metabolites against some cancer types, no study exists investigating the effect of DA on HepG2 cell motility (Kalın et al. 2022a; Günaydın et al. 2023). Our wound healing assay showed that DA possessed an anti-migratory effect against HepG2 cells.

ROS, which are essential for intracellular signaling and regulation of homeostasis, are produced by metabolic activities in healthy cells and eliminated by an enzymatic antioxidant system (Silva et al. 2018; Harris and DeNicola 2020). When there is any problem in this elimination, healthy cells become cancerous (Kirtonia et al. 2020). Interestingly, the activity of enzymatic antioxidant systems also increases in cancer cells, preventing the toxic effect of high ROS and allowing cancer cells to survive (Galadari et al. 2017). Overexpression of TrxR1, which is a member of the Trx antioxidant system, is observed in many tumor types (Raffel et al. 2003; Kim et al. 2003). Therefore, targeting TRXR1 in cancer cells using chemotherapeutic agents is a desirable strategy (Yoo et al. 2006; Ozgencli et al. 2018a, b). Furthermore, in a study performed by Ozgencli et al. (2018a), it was reported that DA may be used as a potential drug for cancer therapy by inhibiting TRXR. For this reason, we investigated the relationship of the anticancer effect of DA with TRXR in HepG2 cells at gene and protein levels and enzymatic activity. Our results showed that although the quantitative gene and protein expression levels of TRXR1 were increased by treatment with DA, TRXR1 activity was significantly decreased. These data suggest that DA may cause post-translational alterations in the TRXR1 enzyme or may be associated with inhibition of the active site of the TRXR1 enzyme. These results are supported by the literature, such that DA affected the TRXR1 gene and protein expressions in lung cancer (A549) and breast cancer (MCF-7 and MDA-MB-453) cell lines, but the actual effect was observed on DA acid has cytotoxic, antimigratory, apoptotic, and necrotic activity in hepatocellular carcinoma (HepG2) cells in vitro. One of the more significant findings of this study is also that the anticancer activity of DA against HepG2 cells is targeted to TRXR1. In conclusion, DA could be accepted as a TRXR1 inhibitor and a potentially useful compound for the treatment of hepatocellular carcinoma.

## Data Availability

The data supporting the findings of the present research are available on request from the corresponding author upon reasonable request.
